# Global economic impacts of climate variability and change during the 20th century

**DOI:** 10.1371/journal.pone.0172201

**Published:** 2017-02-17

**Authors:** Francisco Estrada, Richard S. J. Tol, Wouter J. W. Botzen

**Affiliations:** 1 Centro de Ciencias de la Atmósfera, Universidad Nacional Autónoma de México, Mexico City, Mexico; 2 Institute for Environmental Studies, Vrije Universiteit, Amsterdam, The Netherlands; 3 Department of Economics, University of Sussex, Falmer, United Kingdom; 4 Department of Spatial Economics, Vrije Universiteit, Amsterdam, The Netherlands; 5 Tinbergen Institute, Amsterdam, The Netherlands; 6 CESifo, Munich, Germany; 7 Utrecht University School of Economics (U.S.E.), Utrecht University, Utrecht, The Netherlands; Columbia University, UNITED STATES

## Abstract

Estimates of the global economic impacts of observed climate change during the 20th century obtained by applying five impact functions of different integrated assessment models (IAMs) are separated into their main natural and anthropogenic components. The estimates of the costs that can be attributed to natural variability factors and to the anthropogenic intervention with the climate system in general tend to show that: 1) during the first half of the century, the amplitude of the impacts associated with natural variability is considerably larger than that produced by anthropogenic factors and the effects of natural variability fluctuated between being negative and positive. These non-monotonic impacts are mostly determined by the low-frequency variability and the persistence of the climate system; 2) IAMs do not agree on the sign (nor on the magnitude) of the impacts of anthropogenic forcing but indicate that they steadily grew over the first part of the century, rapidly accelerated since the mid 1970's, and decelerated during the first decade of the 21st century. This deceleration is accentuated by the existence of interaction effects between natural variability and natural and anthropogenic forcing. The economic impacts of anthropogenic forcing range in the tenths of percentage of the world GDP by the end of the 20th century; 3) the impacts of natural forcing are about one order of magnitude lower than those associated with anthropogenic forcing and are dominated by the solar forcing; 4) the interaction effects between natural and anthropogenic factors can importantly modulate how impacts actually occur, at least for moderate increases in external forcing. Human activities became dominant drivers of the estimated economic impacts at the end of the 20th century, producing larger impacts than those of low-frequency natural variability. Some of the uses and limitations of IAMs are discussed.

## Introduction

Integrated assessment models (IAMs) have been widely used for estimating the potential costs of climate change over the 21^st^ and later centuries and for advising policy regarding the desirability of alternative mitigation and adaptation portfolios. However, these models have seldom been applied to the 20th century to examine the impacts of climate change which have already occurred. An exception is Tol[1] who recently applied the FUND model in its national version for estimating the impacts of climate change during the 20th century using observed global temperatures averaged over 5-year periods. His main findings are that while the global average impact over the century was positive, regional and temporal differences are important: most countries benefited from climate change until 1980, but since then the impacts for poor countries have been negative and positive for the rich. The largest negative impacts occur in water and human health.

However, even when filtering out part of the high-frequency variability in observed global temperatures (e.g., by averaging over periods as in Tol[1], running means or filters), the underlying climate change signal is still distorted by the intrinsic low-frequency variability of the climate system, such as long-term oscillations in global temperatures. Moreover, the different contributions of natural and anthropogenic forcing factors to this signal cannot be identified[2–4]. A better understanding of what the economic impacts of the observed climate during the 20th century could have been and of the relative importance of their anthropogenic and natural drivers can provide relevant information for policy-making, socioeconomic research and the society at large. The results presented here are also of interest to the IAM community, as they illustrate the importance of the interaction effects between different impact drivers (i.e., natural variability, natural and anthropogenic forcings). In particular, low-frequency natural variability oscillations can significantly modulate the impacts that would correspond to the observed increases in anthropogenic forcings alone. Depending on their phase and on the magnitude of the interaction effects, the final impacts can be considerably damped or amplified.

Furthermore, a large part of the recent discussion about IAMs has focused on the behavior of their impact functions for large increases in warming and the possible occurrence of catastrophic events[5,6]. Much less attention has been devoted to the uncertainty of these impact functions for small increases in global temperatures, such as observed temperatures in the 20th century or those that are commonly projected to occur during the next few decades. Moreover, the importance of interaction effects of impacts from natural and anthropogenic forcing produced by the nonlinearity of climate impacts has up to our knowledge not been studied yet. Our time-horizon is especially suitable for examining climate change impacts with IAMs impact functions since the observed changes in temperature during this period are well within the limit of 3°C for which these functions have been calibrated. The analyses presented here contribute to the IAMs literature by exploring the multi-model uncertainty for small to moderate increases in warming.

The structure of this paper is as follows. The next section describes the data, scenarios and methods that are used in this study. The third section presents and discusses the estimated costs of climate change over the 20th century and their decomposition in natural and anthropogenic factors. Section four concludes.

## Data and methods

### Climate and radiative forcing databases

We use the HadCRUT3 global surface temperature anomalies time series[7], available at https://figshare.com/s/d8ed9e731989f819d828. We take into account the following indices which are commonly considered to be the most important natural sources of inter-annual global and hemispheric climate variability[8–11]: the Southern Oscillation Index (SOI) from the National Center for Atmospheric Research (NCAR; https://figshare.com/s/d8ed9e731989f819d828) as a proxy for El Niño/Southern Oscillation; the North Atlantic Oscillation (NAO) from Climatic Research Unit (CRU; https://figshare.com/s/d8ed9e731989f819d828); the Atlantic Multidecadal Oscillation (AMO) from the National Oceanic and Atmospheric Administration (NOAA; http://www.esrl.noaa.gov/psd/data/timeseries/AMO/); and the Pacific Decadal Oscillation (PDO) from the Joint Institute for the Study of the Atmosphere and Ocean (JISAO; https://figshare.com/s/d8ed9e731989f819d828).

The radiative forcing series used in this paper are from NASA[12]; available at http://data.giss.nasa.gov/modelforce/). The initial year is 1880 and it is used to represent preindustrial climate forcing, which implies that the values of all radiative forcing variables in that year are zero. We use the following variables (in W/m^2^): well mixed greenhouse gases (RFGHG; carbon dioxide (CO2), methane (NH4), nitrous oxide (N2O); chlorofluorocarbons (CFCs)); tropospheric ozone (O3); stratospheric water vapor; solar irradiance (SOLAR); land use change; snow albedo; black carbon; reflective tropospheric aerosols (RAER) and; the indirect effect of aerosols. As in previous studies[4,13] the total radiative forcing (TRF) is defined as the sum of all the radiative forcing variables mentioned above (both natural and anthropogenic).

### Global temperature scenarios generation

The detection and attribution of climate change has been an area of intense research that has proven to be of interest for a wide range of applications including climate modeling, risk and impact assessment, mitigation and adaptation studies, economics and policy making[14]. The separation of the anthropogenic warming signal from the natural variability in global temperatures has received significant attention during the last decades, leading to the development and adaptation of a variety of statistical and physical modeling approaches to tackle this task[4,13–18]. Although these studies are characterized by strong methodological differences[19], most of them have concluded that global temperature and the total radiative forcing series share a common secular trend. This trend is caused by anthropogenic forcing as a major contributor to the observed warming, and natural variability is characterized as a stationary process.

The existence of this common secular trend allows separating this warming signal from observed global temperature series. For constructing the scenarios used in this paper we apply a simple regression model to detrend observed global temperatures as follows:

$$T_{t}=\alpha +\beta TRF_{t}+u_{t}$$ (1)

**Fig 1:**
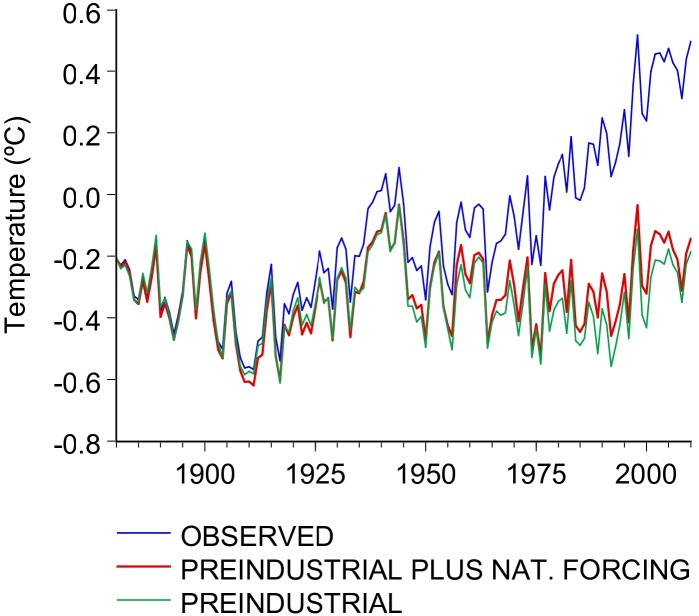
Global temperature scenarios. Observed global temperatures (blue), ${\tilde{t}}_{t}^{*}$ (preindustrial anthropogenic forcing; red) and ${\tilde{t}}_{t}$ (preindustrial forcing; green) for the period 1880–2010.

From which the following quantities can be obtained:

$${\tilde{t}}_{t}=T_{t}-\beta TRF_{t}=\alpha +u_{t}$$ (2)

$${\tilde{t}}_{t}^{*}={\tilde{t}}_{t}+\beta \left(TRF_{t}-RFGHG-RAER\right)$$ (3)

where *T*_*t*_ is the observed global temperature series, *α* is the intercept, *β* is an estimate of the transient climate response[4,20], and *u*_*t*_ are the regression residuals. The coefficients in all three equations are the same and are estimated using the first regression. This simple regression-based method has shown to be adequate for decomposing global temperatures into its anthropogenic and natural components[4,21], although other methods could be used instead[15,22]. Eqs (2) and (3) are used to detrend and partially detrend observed global temperatures, respectively. These time series, depicted in Fig 1, provide alternative climate scenarios as input for running the selected IAMs. The first scenario, ${\tilde{t}}_{t}$ from (Eq 2), represents natural variability under a stationary climate where all external radiative forcings are held constant at their preindustrial values (preindustrial scenario). This preindustrial scenario is similar in concept to the preindustrial control run (piControl) in the Fifth Phase of the Climate Model Intercomparison Project (CMIP5; http://cmip-pcmdi.llnl.gov/cmip5/index.html) conducted for the Fifth Assessment Report of the IPCC[14]. As described by Taylor et al.[23], the climate responds not only to external forcing (attributable both to natural and anthropogenic factors), but it also shows variations that are solely due to internal interactions due to the complex nonlinear climate system. Control runs are carried out to explore this natural “unforced” variability and, for this purpose, all external forcing factors are held at their preindustrial values[14]. The preindustrial values of the external forcing factors are commonly represented by their values on a particular year in the second half the 1800s or some average over this period[23,24]. The second scenario, ${\tilde{t}}_{t}^{*}$ from (Eq 3), represents the evolution of global temperatures holding the main anthropogenic forcing factors (GHG and RAER) constant at their preindustrial values, but allowing all other forcing factors to vary according to the observed records (natural forcing scenario). Note that most of the time-series based attribution studies include only GHG and RAER forcing to represent the observed anthropogenic forcing[4,17,18]. In principle, this approximation could lead to an overestimation of the natural forcing since it excludes only the main anthropogenic forcing. However, the combined radiative forcing of all the other anthropogenic factors (i.e., O3, stratospheric water vapor, land use change, snow albedo, black carbon, and the indirect effect of aerosols) is very small (average value of -0.07W/m^2^) and has practically no effect in the resulting estimated temperatures (the largest difference is -0.05°C). The third scenario, represented by (Eq 1), corresponds to the observed temperature records.

### Impact functions

IAMs are frequently used for advising climate policy and are one of the few available methods for analyzing the economic impacts of climate change at the global level in an internally consistent manner[25,26]. As described below, here we use five impact functions from different IAMs in order to explore the potential consequences that climate change could have already had during the 20^th^ century and to decompose these impacts into their natural and anthropogenic components. Estimating the potential costs of climate change is a challenging task for several reasons. Among the most important are: the wide range of activities, natural and human systems that can be affected by climate change and that need to be included in the assessment of its potential costs[27]; the existence of significant gaps in information, knowledge and methodologies[6,28,29] and; the limited understanding and capacity to model human anticipation and reaction to climate change impacts, such as investments in adaptation[30]. In general, adaptation has been modelled implicitly through the calibration of the impact functions included in the model. Very few exceptions explicitly model adaptation (i.e., AD-DICE[31]). In both cases, adaptation measures are aggregated at the regional level and no explicit microeconomic modeling to represent investment dynamics, and decision making of economic actors is included. As has been shown in the literature, the impacts of climate change can be modified by the agent actions at the micro scale[32–34]. This is one of the most challenging aspects to include in the impact functions of IAMs and contributes to the large uncertainty that characterizes the estimates of the costs of climate change[30]. Given the large complexity of the systems and interactions these models are designed to represent, IAMs are inevitably related with epistemic uncertainty, simplifications and omissions as well as some *ad hoc* and subjective constructs[6,27,29,35–37]. At best, these models can approximate a representation of the current fragmented and incomplete knowledge regarding climate change science and economic impacts from climate change. Furthermore, as has been discussed in the literature, validation and verification of models of complex open systems is problematic and in general model validation and verification can create the misleading illusion that a model is appropriate to support decision-making if its performance for reproducing current observations is deemed to be acceptable[38,39]. Good performance in reproducing the current state of a complex open system is, at best, weakly correlated with better or more reliable projections[40–43]. The economics of climate change, including IAM, faces the additional problem that there is no recorded data regarding the observed welfare impacts of climate change to compare with model outcomes. In fact, if such data would exist then there is no need to estimate past climate impacts using IAMs as we do here. As such, what can be demanded of IAMs is not a model that can reproduce current or past economic states, but that they reasonably represent the state of the knowledge (and uncertainties) about estimating economic impacts of climate change. In the light of these difficulties and those expressed in recent papers[6,29,37], it is important to recall that the primary value of IAMs and other models of complex, open systems is heuristic: they are useful for learning and exploring possible scenarios of how systems can respond to different conditions, but not for producing predictions and, in a strict sense, cannot be validated[38]. Therefore, caution should be exerted when interpreting numerical results of IAMs, as they can give the impression of precision when they are only approximations of how the economic system might respond to climate change that are conditional on a large set of factors and limitations as have been discussed in more detail by other studies[6,27,29].

As noted by several other studies[6,28–30,36], impact functions of IAMs are uncertain because their empirical basis is small. These functions that estimate the GDP consequences for temperature rise are based on statistical and modelling approaches that estimate relations between climate conditions and impacts on a variety of sectors, including: the agricultural sector, coastal areas caused by sea level rise, other market sectors (especially energy use), health risks, immaterial goods (recreation), cities, and ecosystems[44–48]. Moreover, recent literature has focused on estimating the impacts of weather on the economy[49–51] and these results could help providing an empirical foundation for better calibrating and specifying the impact functions in IAMs. However, the impacts from weather shocks and climate change need not be similar and can differ importantly[49,52]. Although some general ideas have been proposed on how to bring climate and weather impacts together, no formal method has been devised to do so. In the present paper, we contribute to this discussion by stressing the existence and importance of interaction effects between natural variability oscillations and the long-term climate signal, which is one aspect needed to estimate the consequences of different changes in climate variables. Moreover, we account for the uncertainty of the impact function by conducting our estimations with a broad range of main impact functions from the IAM literature. The damage functions of IAMs used in this paper come from the most widely used IAMs for estimating the economic costs of climate change[44,47,53–55] and from a meta-analysis review[28,56] that summarizes 21 of such estimates (see S1 Text, section 1). These impact functions are global and no regional versions of impact functions are considered in this study. In what follows the damage functions are denoted as DICE99 and DICE2007, the FUNDn3.6, PAGE2002 and MA (for meta-analysis).

## Results and discussion

In this section we present estimates of the contributions of natural and anthropogenic factors to the estimated costs of observed global temperature during a period comprising the 20th century. Based on the three aforementioned temperature scenarios, five economic impact scenarios are defined:

1. S_OBS: The expected economic costs given the observed global temperature evolution, obtained using *T*_*t*_.
2. S_NV: The expected costs associated with natural variability under a stationary climate holding all external forcing factors constant at their preindustrial levels, obtained using ${\tilde{t}}_{t}$.
3. S_NVF: The expected costs associated with the observed natural external forcing and internal variability, obtained using ${\tilde{t}}_{t}^{*}$. This scenario is used only for estimating S_AF and S_NF described below.
4. S_AF: The expected costs associated with the anthropogenic radiative forcing, obtained as the difference of S_OBS and S_NVF.
5. S_NF: The expected costs associated with the natural radiative forcing, obtained as the difference of S_NVF and S_NV.

Note that the impact scenarios above are composed of the combination of the contributions of natural and anthropogenic factors. Given the nonlinear functional forms in the impact functions used, interaction effects between the different components are produced. Consider as an illustration *D* = *f*(*a* + *b*), where *f* is, for example, a quadratic function. In this case, *D* would be equal to the sum of *a*^2^+*b*^2^, plus the interaction term 2*ab*. The approach for separating the contributions of internal variability and anthropogenic and natural forcing described in steps 1 to 5 above preserves their interaction effects (e.g., the effects of natural variability under a stationary climate are not the same than under an externally forced climate due to the nonlinearities in the damage functions).

### Estimates of costs from observed global temperatures

**Fig 2:**
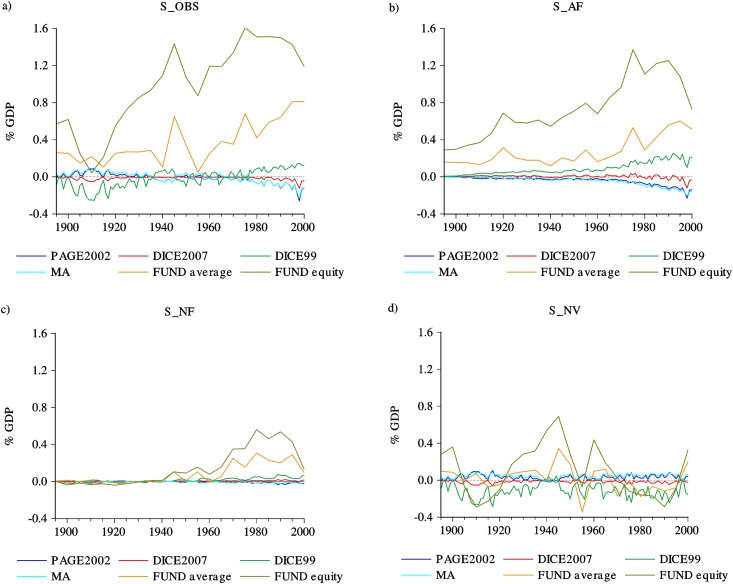
Economic effects over the 20th century according to different damage functions. Panels show (a) the economic impacts of observed temperature (S_OBS), (b) the economic impacts associated with the effects of anthropogenic radiative forcing (S_AF), (c) the economic impacts associated with the effects of natural radiative forcing (S_NF) and (d) the economic impacts associated with the effects of natural variability (S_NV).

Panel a) of Fig 2 shows the estimated impacts of the observed climate during the 20th century obtained from the 5 different IAMs impact functions. According to PAGE2002, MA and DICE2007, by the end of the century the observed global temperature had a negative effect on welfare. For DICE99 and FUNDn3.6 the effect was positive. While DICE99, DICE2007, MA and PAGE2002 suggest that the economic impacts during the last decade are small (about -0.26% to 0.14% of global GDP), FUNDn3.6 shows considerably larger (positive) impacts reaching about 0.8% of GDP in 2000. FUNDn3.6 equity weighting results show the highest benefits: 1.19% in 2000 and a maximum of 1.61% in the mid-1970s. According to the FUND model during the 20th century the poorer countries experienced greater benefits, primarily from CO2 fertilization, than the richer countries and therefore the equity weighted impacts are more positive than the non-weighted average[28]. The differences in the projected impacts mainly arise from small differences in included climate impact categories[28,56] and from differences in how the impact functions are specified. In particular, the chosen functional form for the impact functions has an important effect over the projected impacts and these can vary greatly from model to model: while the functional form in DICE1999, DICE2007 and MA is quadratic, in PAGE2002 the functional form goes from linear to cubic, and in FUND each sector has specific functional forms.

**Fig 3:**
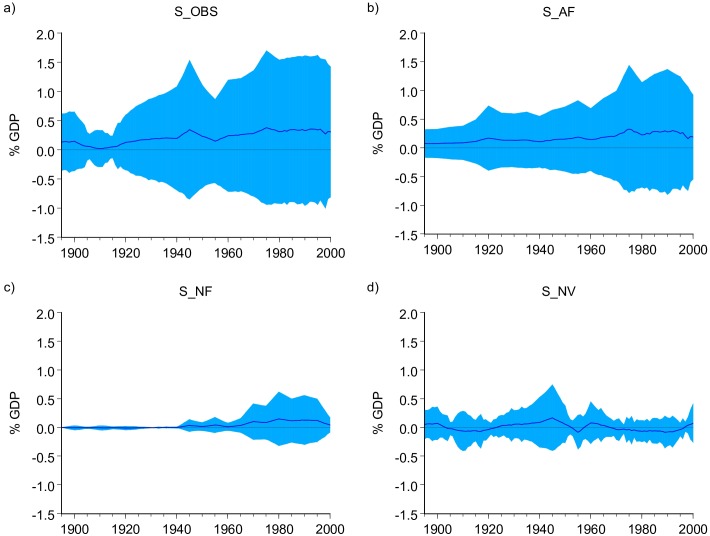
Multimodel mean of the estimated economic effects over the 20th century. Multimodel estimates of the economic impacts of observed global temperature (S_OBS), (b) the economic impacts associated with the effects of anthropogenic radiative forcing (S_AF), (c) the economic impacts associated with the effects of natural radiative forcing (S_NF) and (d) the economic impacts associated with the effects of natural variability (S_NV).

With the exception of PAGE, all other impact functions used in this paper are deterministic and do not provide information regarding the uncertainty in the estimated costs. Nevertheless, by using all the estimates produced by the individual impact functions a general uncertainty interval can be calculated. Fig 3 panel a) shows the multimodel mean of S_OBS and the corresponding two standard deviation intervals representing the uncertainty in this estimate. The multimodel mean in Fig 3 panel a) shows a steady positive trend that leads to net benefits of about 0.30% of GDP in 2000. Note however that throughout the 20th century, the multimodel mean value is always smaller than the standard deviation of the models' outcomes, underlying the very large uncertainty in these estimates (e.g., the standard deviation in 2000 was 0.56%). For the estimates in Fig 3 all IAMs are weighted equally, implying that all of them produce equally credible estimates.

### Contributions of the natural and anthropogenic radiative forcing to the estimated impacts

Panels a), b) and c) of Fig 2 show that the trending behavior of the estimated global economic impacts S_OBS can only be produced by S_AF and S_NF which share a somewhat similar nonlinear trend. However, the magnitude of the impacts produced S_NF is, for most models, about one order of magnitude lower than those associated with anthropogenic forcing. As clearly shown in panel d), the costs associated with natural variability describe oscillatory patterns around a fixed mean that cannot account for the trend in global impacts.

According to PAGE2002, MA, DICE99 and DICE2007, the welfare impacts of anthropogenic forcing lie in the range of a few tenths of percent of the world GDP by the end of the 20th century (from -0.23% in PAGE2002 to 0.24% in DICE99). This figure is considerably larger for FUNDn3.6 which indicates benefits in the range of about 0.60% to 1.37%. It is also worth noting that DICE2007 provides the smallest estimates of impacts, reaching only about -0.1% at the end of the century.

**Fig 4:**
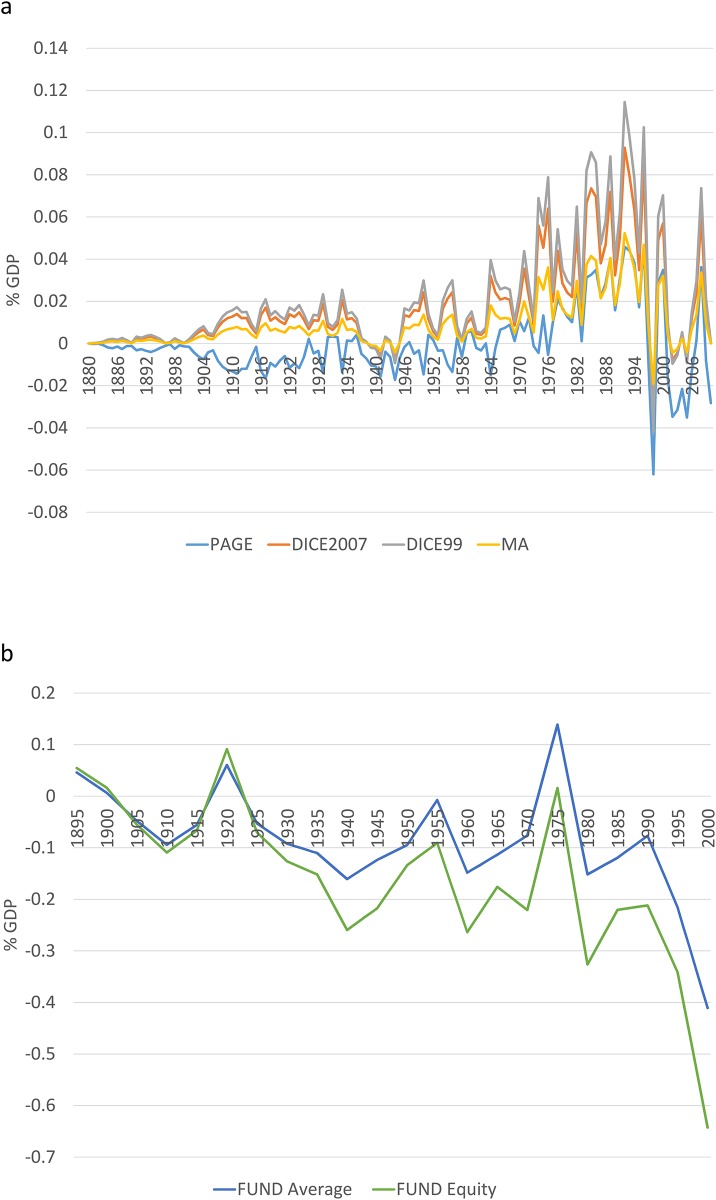
Interaction effects for the economic impacts of anthropogenic forcing. (a) interaction effects for PAGE, DICE2007, DICE99 and MA. (b) interaction effects for FUNDn3.6 average and FUNDn3.6 equity. NI denotes that interaction effects are not included.

It is of particular interest to quantify the interaction effects produced by the different components of global temperatures. The implicit assumption in IAM applications is that the estimation of the economic costs of climate change can be based on stylized temperature projections based only on anthropogenic forcing; i.e., economic impacts are linearly separable into their components caused by different kinds of forcing. As illustrated below, this assumption does not hold and can considerably bias the impact estimates. Fig 4 shows the interaction effects, obtained as the difference of S_AF and the costs estimated using the temperature based on anthropogenic forcing only (S4 Fig). These interaction effects are characterized by a nonlinear trend that depends on the magnitude of anthropogenic forcing, natural forcing and variability and on the particular specification of the impact function. These synergistic impacts have non-negligible magnitudes, get larger as the observed anthropogenic forcing increases and can significantly change the evolution of impacts. The amplitudes of the interaction effects ranges from 0.07% (MA) to 0.16% (DICE99) of GDP, and in the case of FUND the amplitudes are 0.55% (average) and 0.73% (equity) of GDP. For all of the impact functions, the magnitude of the interaction effects is comparable to, or are larger than, those of S_NF. The slowdown in the anthropogenic radiative forcing experienced since the early 1990’s provides an illustration of how much these interaction effects can modify the estimated impacts. Since the last years of the 1990s, the estimated impacts decreased in magnitude which is in part due to the aforementioned slowdown. However, as shown by S4 Fig, this reduction was heavily reinforced by the interaction effects, leading to a significant drop in the magnitude of the estimated impacts since the late 1990s.

The multimodel mean of S_AF indicates that the human contribution to the observed warming during the 20th century produced net benefits in the world average. The benefits increased from about 0.08% at the beginning of the century to about 0.19% of GDP in 2000 after reaching about 0.33% in the 1990's (Fig 3 panel b). As before, the uncertainty is quite large: the multimodel mean is always smaller than the standard deviation of the models' outcomes.

The contribution of S_NF to the overall impacts is depicted in panel c) of Fig 2. The magnitude of the impacts is considerably lower than that of S_AF, amounting to at most 0.1% during the century, with the exception of FUNDn3.6 in which the highest values of S_NF are in the range of 0.3% to 0.5%. With the exception of DICE2007, the increases in natural forcing observed since the mid-20th century make S_NF contribute in the same direction as S_AF to the estimated total costs. This is consistent with climate physics: irrespective of their origin, increases in radiative forcing simply add up, leading to larger climate transient response and equilibrium temperatures[20]. The effects of natural forcing are dominated by the eleven-year cycle in solar forcing. The correlation between the impacts attributed to natural forcing factors with solar forcing is very large and positive for DICE99, DICE2007, MA and FUNDn3.6 ranging from 0.62 to 0.91, while for PAGE2002 this correlation is -0.84.

The multimodel mean shows that the impacts of S_NF where practically zero until the 1940s. In the second half of the century natural forcing (mainly solar) produced small but increasing benefits reaching around 0.04% of GDP in 2000 (Fig 3c).

### Estimates of costs obtained from the preindustrial scenario

All of the impact functions indicate that the natural variability alone can lead to impacts that are comparable in magnitude to those that can be attributed to anthropogenic factors until the last three decades of the 20th century, and are much larger than those that can be associated with the observed natural forcing (Fig 2d). The main difference is that the natural variability impacts follow low-frequency oscillations instead of sustained trends. The impacts under the preindustrial scenario can be associated with some of the main modes of interannual climate variability. As shown in S3 Table, S_NV is highly and significantly correlated with AMO and to a lesser extent with SOI, PDO and NAO. The magnitude of these correlations is broadly similar for the estimates obtained using the PAGE2002, MA, DICE99 and DICE2007 impact functions (about 0.70, 0.30, 0.20 and 0.24 in absolute value for AMO, SOI, PDO and NAO, respectively), although the signs are different depending on the specification of the impact functions. Only in the case of DICE2007 the impacts of natural variability are strictly negative, while for DICE99 they are mostly negative and for PAGE2002 and MA they are mainly positive. These non-monotonic impacts are dominated by the low-frequency variability and large persistence of the climate system.

Linear regression models using AMO, SOI, PDO and NAO as explanatory variables were estimated, but only the first two (AMO and SOI) were found to significantly contribute to explain the variability of the estimated costs. The following specification was found to be statistically adequate for most of the IAMs estimates (see S4 and S5 Tables for parameter estimates and misspecification tests):

$$S\_NV_{it}=c+\alpha S\_NV_{it-1}+\delta_{1}AMO_{t}+\delta_{2}AMO_{t-1}+\gamma SOI_{t}+\varepsilon_{t}$$ (4)

where *S_NV*_*it*_ are the estimated costs for model *i* = 1,…,5. This regression model has a similar specification to those in previous studies[4] for global temperature series. In all cases AMO and SOI are highly significant, except for the estimates obtained with FUNDn3.6 where only AMO is significant.

For most IAMs, the estimated regressions explain about 60% of the variance of the impacts associated with natural variability. Furthermore, AMO and SOI generate important fluctuations from the mean of *S_NV*_*it*_: a one standard deviation shock to AMO produces a cumulative long-run response of about 0.60 times the standard deviation of *S_NV*_*it*_ (positive for DICE99 and DICE2007, negative for PAGE2002 and MA) while a shock of one standard deviation to SOI generates a long-term response 0.45 times the standard deviation of *S_NV*_*it*_ (negative for DICE99 and DICE2007, the opposite occurs with PAGE2002. See S6 Table). For FUNDn3.6 a one standard deviation shock in AMO produces a response of 0.39 (average) and 0.77 (equity) times the standard deviation of *S_NV*_*it*_. These long-run responses are calculated by scaling the coefficients of the explanatory variables in (Eq 4) by 1/(1-α).

The multimodel mean of S_NV is mainly negative and shows a low-frequency oscillatory pattern similar to AMO (correlation coefficient of 0.60) varying in a range of -0.08% to 0.17% of GDP during the 20th century. It is worth noticing that the standard deviation of the models' outcome is on average almost 3 times larger than the multimodel mean, indicating the large uncertainty in this estimate. Furthermore, S_NV shows that until the last three decades of the 20th century, natural variability was the main source of economic impacts. Since then, the main driver of impacts is anthropogenic forcing.

### Sectoral decomposition of impacts

According to the sectoral decomposition of the estimated impacts obtained by FUNDn3.6 (S1 Text, section 2), anthropogenic forcing in agriculture accounts for most of the economic benefit in the past century (S1 Fig). Benefits attributable to the anthropogenic forcing are also found for the energy sector, while this forcing imparted a trend in the economic losses in human health and water resources. The model strongly suggests that the contribution of anthropogenic forcing to the estimated number of deaths per thousand people is dominant in the case of diarrhoea, respiratory diseases and malaria (S2 Fig).

## Discussion

This paper adds to the recent discussion regarding IAMs by investigating the differences in the estimates obtained from model to model for small increases in temperatures. Even though the estimates of the global economic impacts of climate change used as benchmarks to calibrate IAMs are in broad agreement[28,56], IAMs impact functions do not agree in the sign nor the magnitude of the impacts for small changes in temperature (S3 Fig). These differences are largely due to how the impact function is specified, in particular the functional form that is chosen and if the dynamics of impacts are modeled[5,30]. In the case of FUNDn3.6 and DICE99 the observed warming has brought benefits to global welfare, while according to DICE2007, MA and PAGE2002 the opposite is true. With the exception of FUNDn3.6, which estimates the magnitude of the impacts in about 1% of GDP at the end of the 20th century, the rest of the IAMs considered value the impacts in only a few tenths of percent.

Despite the uncertainty in impact functions estimates, some robust results are obtained. First, the magnitude of the impacts over the last three decades is unprecedented over the last century. Only in the case of DICE99 the magnitude of the impacts attributable to natural variability are larger than those of the anthropogenic forcing at end of the 20th century. Second, the decomposition of the estimated impacts of observed global temperature reveals that at the end of the 20th century anthropogenic forcing became the dominant driver of the estimated economic impacts, producing similar or larger impacts than those of low-frequency natural variability. Anthropogenic impacts increased over the period of analysis in a non-monotonic way, slowly for the first part of the 20th century, accelerating significantly after the 1970s and reducing their rate of increase after the 1990s when a slowdown in global warming started[4,13,57,58]. Third, it is shown that the interaction effects can notably modulate the estimates of the economic impacts of climate change. If these effects are not considered as is common practice, the estimated costs of climate change can be biased. Fourth, the contribution of natural forcing to the total estimated impacts is about one order of magnitude lower than that of the anthropogenic forcing or that of the internal interannual variability. The main driver of the impacts associated with natural factors is solar forcing, which imprinted its 11-year cycle and a slight positive trend. Fifth, in the intra- and inter-decadal scales the amplitude of the impacts associated with natural variability is considerably larger than that produced by anthropogenic factors during the first half of the century. These non-monotonic impacts are mostly determined by the low-frequency variability modes and persistence of the climate system.

## Conclusion

As is common in climate change science and modeling, IAMs have important limitations and are fraught with uncertainty. Nevertheless, these models are valuable tools for supporting decision making and for exploring the potential economic consequences of climate change. This paper illustrates the large uncertainty in the impact functions projections for small increases in warming, such as that of the observed warming period and those that are projected to occur in the short- and medium-terms. Given the common use of positive discount rates, the impacts in the near and medium future can have a significant weight on the present value estimates of climate change costs. Investigating the differences in IAMs impact functions and improving their calibration for small increases in warming would help providing better estimates of the economic costs of climate change. The results of this paper point to the importance of interaction effects which are currently ignored in IAMs projections of the costs of future climate change. Most IAMs produce temperature projections based exclusively on anthropogenic forcing, implicitly assuming that the different natural and anthropogenic contributions to the climate change costs are linearly separable. Given the nonlinearity of impact functions this is not the case and as is shown in this paper the interaction effects can be large, potentially biasing the estimates if ignored. The consequences of this assumption for the estimates of future climate change costs will be addressed by the authors in a forthcoming paper.

## Supporting information

S1 Fig Estimated economic effects over the 20th century per sector. (a) agriculture, (b) water resources, (c) energy and (d) health.
(TIF)

S2 Fig Estimated deaths per million people during the 20th century per disease. Deaths caused by (a) impacts associated to observed global temperature change (S_OBS), (b) impacts associated to the effects of anthropogenic radiative forcing (S_AF), (c) impacts associated to the effects of natural radiative forcing (S_NF) and (d) impacts associated to the effects of natural variability (S_NV).
(TIF)

S3 Fig Estimated economic effects for the 20th century per IAM. (a) PAGE2002, (b) DICE99, (c) DICE2007, (d) MA, (e) FUNDn3.6 average and (f) FUNDn3.6 equity.
(TIFF)

S4 Fig Estimated economic effects due to anthropogenic forcing with and without interaction effects. (a) estimates for PAGE, DICE2007, DICE99 and MA. (b) estimates for FUNDn3.6 average and FUNDn3.6 equity. NI denotes that interaction effects are not included.
(TIF)

S1 Table Parameter values of the damage functions in the DICE99 and DICE2007 models. (DOCX)

S2 Table Parameter values for the economic and non-economic sectors for EU and regional weights from PAGE2002. (DOCX)

S3 Table Correlation coefficients between the estimated impacts from the preindustrial scenario and AMO, SOI, NAO and PDO. (DOCX)

S4 Table Regression models for *S_NV*_*it*_ based on key variability modes and the persistence of impacts. (DOCX)

S5 Table Misspecification testing for the models for *S_NV*_*it*_ based on key variability modes and the persistence of impacts. (DOCX)

S6 Table Long-run response of estimated impacts to one standard deviation shocks to AMO and SOI as a percentage of GDP. (DOCX)

S1 Text Supplementary information about impact functions and additional results. (DOC)
