# Supplementary figures and images for: Global economic impacts of climate variability and change during the 20th century
Source: PLoS One. 2017 Feb 17;12(2):e0172201. doi: 10.1371/journal.pone.0172201 (PMC5315296; doi:10.1371/journal.pone.0172201)

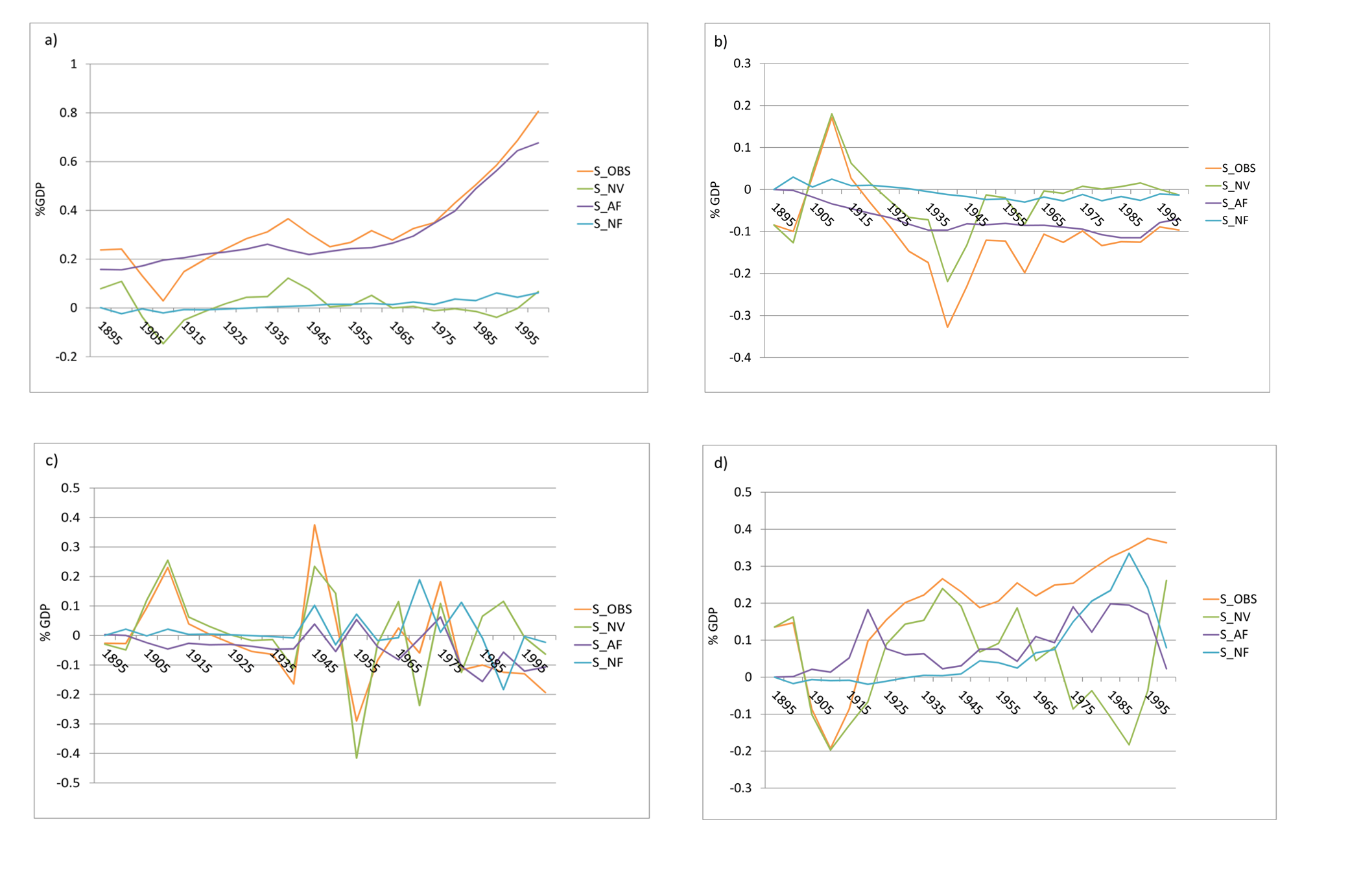

Supplement: S1 Fig — (a) agriculture, (b) water resources, (c) energy and (d) health. (TIF) [file pone.0172201.s001.tif]

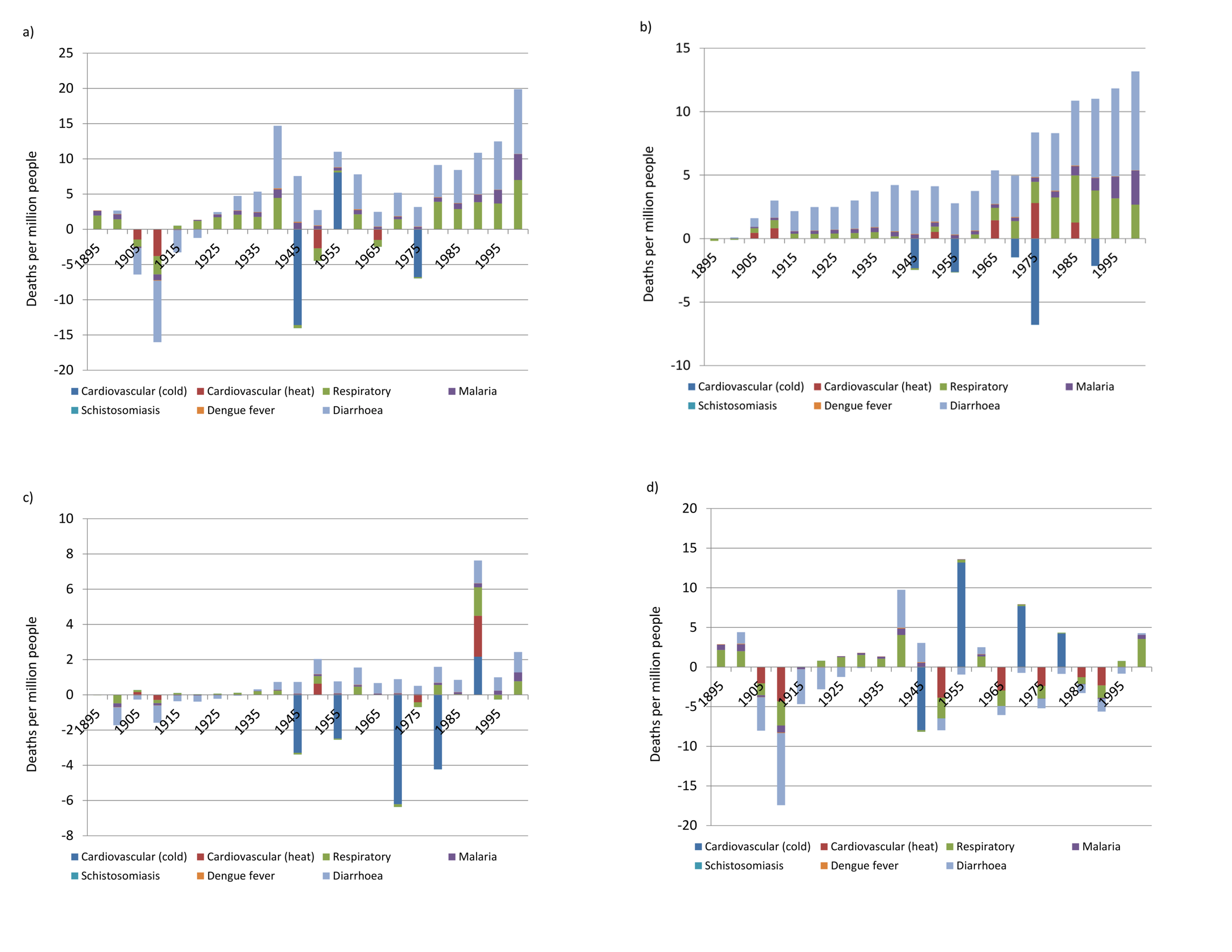

Supplement: S2 Fig — Deaths caused by (a) impacts associated to observed global temperature change (S_OBS), (b) impacts associated to the effects of anthropogenic radiative forcing (S_AF), (c) impacts associated to the effects of natural radiative forcing (S_NF) and (d) impacts associated to the effects of natural variability (S_NV). (TIF) [file pone.0172201.s002.tif]

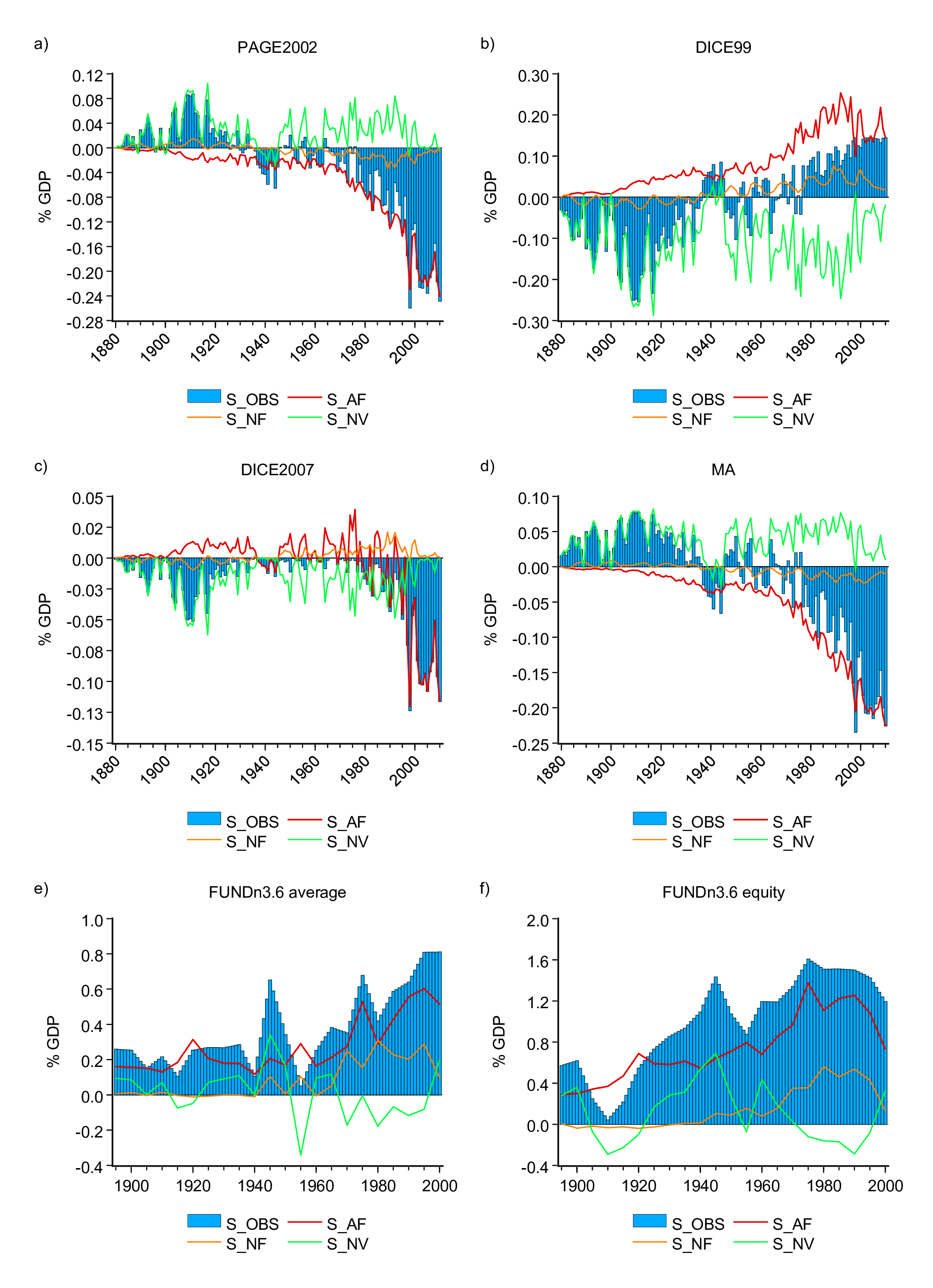

Supplement: S3 Fig — (a) PAGE2002, (b) DICE99, (c) DICE2007, (d) MA, (e) FUNDn3.6 average and (f) FUNDn3.6 equity. (TIFF) [file pone.0172201.s003.tiff]
