# Supplementary material for: Global economic impacts of climate variability and change during the 20th century
Source: PLoS One. 2017 Feb 17;12(2):e0172201. doi: 10.1371/journal.pone.0172201 (PMC5315296; doi:10.1371/journal.pone.0172201)
Supplement: S1 Table — (DOCX) [file pone.0172201.s005.docx]

**Table S1. Parameter values of the damage functions in the DICE99 and DICE2007 models.**

| Model | $\theta_{1}$ | $\theta_{2}$ |
| --- | --- | --- |
| DICE99 | -0.00450 | 0.00350 |
| DICE2007 | 0.00000 | 0.00284 |
