# Supplementary material for: Global economic impacts of climate variability and change during the 20th century
Source: PLoS One. 2017 Feb 17;12(2):e0172201. doi: 10.1371/journal.pone.0172201 (PMC5315296; doi:10.1371/journal.pone.0172201)
Supplement: S2 Table — (DOCX) [file pone.0172201.s006.docx]

**Table S2. Parameter values for the economic and non-economic sectors for EU and regional weights from PAGE2002.**

|  | Mean | Min | Mode | Max |
| --- | --- | --- | --- | --- |
| Economic impact in EU (%GDP for 2.5°C) | 0.5 | -0.1 | 0.6 | 1 |
| Non-economic impact EU (%GDP for 2.5°C) | 0.73 | 0 | 0.7 | 1.5 |
| Impact function exponent | 1.76 | 1 | 1.3 | 3 |
| Eastern Europe & FSU weights factor | -0.35 | -1 | -0.25 | 0.2 |
| USA weights factor | 0.25 | 0 | 0.25 | 0.5 |
| China weights factor | 0.2 | 0 | 0.1 | 0.5 |
| India weights factor | 2.5 | 1.5 | 2 | 4 |
| Africa weights factor | 1.83 | 1 | 1.5 | 3 |
| Latin America weights factor | 1.83 | 1 | 1.5 | 3 |
| Other OECD weights factor | 0.25 | 0 | 0.25 | 0.5 |
