# Supplementary material for: Global economic impacts of climate variability and change during the 20th century
Source: PLoS One. 2017 Feb 17;12(2):e0172201. doi: 10.1371/journal.pone.0172201 (PMC5315296; doi:10.1371/journal.pone.0172201)
Supplement: S3 Table — (DOCX) [file pone.0172201.s007.docx]

**Table S3. Correlation coefficients between the estimated impacts from the preindustrial scenario and AMO, SOI, NAO and PDO.**

|  | DICE99 | DICE2007 | MA | PAGE2002 | FUND average | FUND equity |
| --- | --- | --- | --- | --- | --- | --- |
| AMO | 0.70  (0.000) | -0.65  (0.000) | -0.70  (0.000) | -0.68  (0.000) | 0.38  (0.097) | 0.77  (0.000) |
| NAO | -0.24  (0.012) | 0.23  (0.017) | 0.24  (0.012) | 0.24  (0.011) | 0.16  (0.506) | -0.19  (0.428) |
| SOI | -0.30  (0.002) | 0.31  (0.001) | 0.28  (0.004) | 0.31  (0.001) | 0.18  (0.452) | 0.10  (0.673) |
| PDO | 0.21  (0.025) | -0.18  (0.061) | -0.23  (0.015) | -0.20  (0.032) | -0.05  (0.829) | -0.06  (0.817) |

P-values in parenthesis.
