# Supplementary material for: Global economic impacts of climate variability and change during the 20th century
Source: PLoS One. 2017 Feb 17;12(2):e0172201. doi: 10.1371/journal.pone.0172201 (PMC5315296; doi:10.1371/journal.pone.0172201)
Supplement: S4 Table — (DOCX) [file pone.0172201.s008.docx]

**Table S4. Regression models for $S\_NV_{it}$ based on key variability modes and the persistence of impacts.**

| $S\_NV_{it}$ | c | $\alpha$ | $\delta_{1}$ | $\delta_{2}$ | $\gamma$ | $R^{2}$ |
| --- | --- | --- | --- | --- | --- | --- |
| DICE99 | -0.0638  **(-7.41)** | 0.4147  **(5.55)** | 0.2500  **(8.27)** | -0.1041  **(-2.92)** | -0.0174  **(-4.75)** | 0.65 |
| DICE2007 | -0.0099  **(-7.06)** | 0.3632  **(4.66)** | 0.0463  **(6.87)** | -0.0169  **(-2.24)** | -0.0374  **(-4.61)** | 0.58 |
| MA | 0.0211  **(-6.34)** | 0.4721  **(6.04)** | -0.0915  **(-9.34)** | 0.0518  **(4.46)** | **--** | 0.62 |
| PAGE2002 | 0.0160  **(6.79)** | 0.3941  **(5.21)** | -0.0925  **(-7.83)** | 0.0387  **(2.85)** | 0.0070  **(4.88)** | 0.63 |
| FUND average | 0.0311  (1.01) | -- | 0.3917  *(1.89)* | -- | -- | 0.15 |
| FUND equity | 0.1595  **(3.91)** | -- | 1.4678  **(5.35)** | -- | -- | 0.59 |

Bold and italic figures indicate statistical significance at the 5% and 10 levels. t-statistics are given in parenthesis.
