# Supplementary material for: Global economic impacts of climate variability and change during the 20th century
Source: PLoS One. 2017 Feb 17;12(2):e0172201. doi: 10.1371/journal.pone.0172201 (PMC5315296; doi:10.1371/journal.pone.0172201)
Supplement: S5 Table — (DOCX) [file pone.0172201.s009.docx]

**Table S5. Misspecification testing for the models for $S\_NV_{it}$ based on key variability modes and the persistence of impacts.**

| Misspecification test | DICE99 | DICE2007 | PAGE2002 | MA | FUND average | FUND equity |
| --- | --- | --- | --- | --- | --- | --- |
| RESET  (F-statistic) |  |  |  |  |  |  |
| 1 | 0.399  (0.529) | 1.887  (0.062) | 0.149  (0.700) | 1.498  (0.137) | 0.545  (0.469) | 1.028  (0.317) |
| 2 | 0.218  (0.805) | 2.355  (0.099) | 0.077  (0.926) | 1.114  (0.332) | 0.311  (0.737) | 1.096  (0.355) |
| 3 | 0.245  (0.865) | 2.123  (0.101) | 0.078  (0.972) | 1.041  (0.377) | 0.241  (0.866) | 0.692  (0.569) |
| 4 | 0.590  (0.670) | 2.117  (0.083) | 0.911  (0.460) | 1.003  (0.409) | 0.401  (0.805) | 0.602  (0.667) |
| Jarque-Bera |  |  |  |  |  |  |
|  | 1.064  (0.588) | 6.654  **(0.036)** | 2.904  (0.234) | 1.483  (0.476) | 9.54  **(0.009)** | 0.509  (0.775) |
| Ljung-Box  (Q-statistic) |  |  |  |  |  |  |
| 1 | 0.094  (0.759) | 0.023  (0.879) | 0.016  (0.900) | 0.123  (0.726) | 0.523  (0.470) | 2.564  (0.109) |
| 2 | 1.813  (0.404) | 3.877  (0.144) | 3.122  (0.210) | 0.504  (0.777) | 2.809  (0.246) | 4.595  (0.101) |
| 3 | 1.825  (0.609) | 3.928  (0.269) | 3.140  (0.371) | 0.829  (0.843) | 5.270  (0.153) | 6.151  (0.104) |
| 4 | 7.631  (0.106) | 8.099  (0.088) | 8.539  (0.074) | 3.876  (0.423) | 5.324  (0.256) | 6.152  (0.188) |
| White (F-statistic) |  |  |  |  |  |  |
|  | 1.252  (0.249) | 1.620  (0.084) | 1.580  (0.095) | 1.230  (0.283) | 0.724  (0.498) | 0.442  (0.649) |
| McLeod-Li |  |  |  |  |  |  |
| 1 | 0.064  (0.800) | 2.857  (0.091) | 1.080  (0.299) | 1.404  (0.236) | 0.359  (0.549) | 0.889  (0.346) |
| 2 | 0.193  (0.908) | 4.408  (0.110) | 1.334  (0.513) | 1.520  (0.468) | 2.530  (0.282) | 2.180  (0.336) |
| 3 | 0.224  (0.974) | 7.485  (0.058) | 2.430  (0.488) | 1.698  (0.637) | 2.610  (0.456) | 2.569  (0.463) |
| 4 | 0.708  (0.950) | 7.743  (0.101) | 2.664  (0.616) | 1.741  (0.783) | 3.099  (0.541) | 3.238  (0.519) |
| Breusch-Godfrey  (F-statistic) |  |  |  |  |  |  |
| 1 | 0.277  (0.599) | 0.081  (0.777) | 0.048  (0.827) | 0.525  (0.470) | 0.417  (0.526) | 2.184  (0.156) |
| 2 | 2.161  (0.120) | 2.564  (0.081) | 2.730  (0.069) | 0.281  (0.756) | 1.299  (0.297) | 3.288  (0.061) |
| 3 | 1.481  (0.223) | 1.700  (0.171) | 1.834  (0.145) | 0.299  (0.826) | 1.230  (0.330) | 2.071  (0.142) |
| 4 | 2.363  (0.057) | 1.901  (0.115) | 2.328  (0.060) | 1.041  (0.389) | 0.880  (0.498) | 1.492  (0.251) |
| ARCH  (F-statistic) |  |  |  |  |  |  |
| 1 | 0.062  (0.804) | 2.803  (0.097) | 1.046  (0.309) | 1.360  (0.246) | 0.278  (0.605) | 0.713  (0.409) |
| 2 | 0.091  (0.913) | 1.861  (0.160) | 0.582  (0.560) | 0.800  (0.451) | 0.864  (0.439) | 0.679  (0.520) |
| 3 | 0.082  (0.970) | 1.907  (0.132) | 0.668  (0.574) | 0.590  (0.623) | 0.502  (0.686) | 0.433  (0.732) |
| 4 | 0.174  (0.951) | 1.637  (0.169) | 0.569  (0.686) | 0.434  (0.784) | 0.536  (0.712) | 0.581  (0.682) |
| CUSUM | Stability | Stability | Stability | Stability | Stability | Stability |
| CUSUMQ | Stability | Stability | Stability | Stability | **Instability** | Stability |

Bold figures indicate statistical significance at the 5% level. P-values are given in parenthesis.
