# Supplementary material for: Global economic impacts of climate variability and change during the 20th century
Source: PLoS One. 2017 Feb 17;12(2):e0172201. doi: 10.1371/journal.pone.0172201 (PMC5315296; doi:10.1371/journal.pone.0172201)
Supplement: S6 Table — (DOCX) [file pone.0172201.s010.docx]

**Table S6. Long-run response of estimated impacts to one standard deviation shocks to AMO and SOI as a percentage of GDP.**

|  | DICE99 | DICE2007 | MA | PAGE2002 | FUND average | FUND equity |
| --- | --- | --- | --- | --- | --- | --- |
| AMO | 0.046  [0.635] | -0.009  [-0.587] | -0.014  [-0.598] | -0.017  [-0.602] | 0.058  [0.390] | 0.216  [0.767] |
| SOI | -0.033  [-0.451] | 0.007  [0.444] | -- | 0.014  [0.470] | -- | -- |

Numbers in brackets represent the response of the estimated impacts as a fraction of their standard deviation.
