# Supplementary material for: Global economic impacts of climate variability and change during the 20th century
Source: PLoS One. 2017 Feb 17;12(2):e0172201. doi: 10.1371/journal.pone.0172201 (PMC5315296; doi:10.1371/journal.pone.0172201)
Supplement: S1 Text — (DOC) [file pone.0172201.s011.doc]

Global economic impacts of climate variability and change during the 20^th^ century.

Short title: Economic impacts of climate variability and change

Francisco Estrada^1,2*^, Richard S.J. Tol^3,2,4,5,6^ and Wouter J.W. Botzen ^2,7^

^1^ Centro de Ciencias de la Atmósfera, Universidad Nacional Autónoma de México, Mexico City, Mexico

^2^ Institute for Environmental Studies, Vrije Universiteit, Amsterdam, The Netherlands

^3^ Department of Economics, University of Sussex, Falmer, United Kingdom

^4^ Department of Spatial Economics, Vrije Universiteit, Amsterdam, The Netherlands

^5^ Tinbergen Institute, Amsterdam, The Netherlands

^6^ CESifo, Munich, Germany

^7^ Utrecht University School of Economics (U.S.E.), Utrecht University, Utrecht, The Netherlands.

^*^ Corresponding author

[feporrua@atmosfera.unam.mx](mailto:feporrua@atmosfera.unam.mx) (FE)

S1 Text. Supplementary information about impact functions and additional results.

Content

1. Description of damage functions from IAMs

1.1. FUND model description

1.2. The DICE damage function

1.3. The PAGE2002 damage function

1.4. Damage function from recent literature review

2. Sectoral estimates using FUND

**1. Description of damage functions from IAMs**

**1.1. FUND model description**

In this paper we apply the national version of the Climate Framework for Uncertainty, Negotiation and Distribution (FUNDn3.6;[1]). In contrast to other versions of the model, which endogenously generate scenarios for population, economy, energy use and emissions and include a simple carbon cycle and climate model, this version of FUND is limited to the impacts of climate change. The impact module includes the following categories: agriculture, forestry, sea level rise, cardiovascular and respiratory disorders related to cold and heat stress, malaria, dengue fever, schistosomiasis, diarrhoea, energy consumption, water resources, unmanaged ecosystems, and tropical and extra tropical storms[2,3]. The model estimates the climate related damages attributed to either the rate of change or to the level of change with damages slowly fading due to autonomous adaptation. This version of the model runs in 5-year time steps[3,4] and the reference temperature value is the average of the 1961-1990 period. For a more detailed description of the model, the reader is referred to the original papers and technical documentation available at <http://www.fund-model.org/>; the model code for this version is available at http://dvn.iq.harvard.edu/dvn/dv/rtol.

**1.2. The DICE damage function**

The damage function of DICE was developed from estimates from 12 world regions and includes damages to major sectors such as agriculture, the cost of sea-level rise, adverse impacts on health, and nonmarket damages, as well as estimates of the potential costs of catastrophic damages[5]. The aggregated impact function can be described as follows:

$D_{t}=\theta_{1}T_{t}+\theta_{2}T_{t}^{2}$ (4)

where $D_{t}$ represents the climate damage as fraction of output, $\theta_{1}$ and $\theta_{2}$ are the parameters of the damage function calibrated for the world, $T_{t}$ is global temperature increase over its 1900 value. For this paper we consider the DICE99[6] and the DICE2007[5,7]. Parameterizations are shown in S1 Table. The main difference in the parameterization of DICE99 and DICE2007 consists in that in the former the climate impacts for small temperature increases were estimated to produce net positive benefits, while in the latter all temperature increases lead to net negative impacts[5]. A one-year time step was chosen for all estimates presented here.

**1.3. The PAGE2002 damage function**

The PAGE2002 model damage functions include the uncertainty in the functions' coefficients by means of triangular distributions parameterized to cover the range of possible impacts that have been reported in the literature. The main aim is to offer a probabilistic representation of the potential climate change damages to inform decision-making[8].

The impact functions of PAGE2002 can be expressed as follows:

$I_{t,d,r}=\alpha_{d,r}\left(\frac{\Delta T_{t,r}}{2.5}\right)^{\beta}Y_{t,r}$ (5)

$D_{t,r}=\gamma_{t,r}\pi_{t}Y_{t,r}$ (6)

where $I_{t,d,r}$ represents the economic impacts in time *t*, in the sector *d* (*d=1,2*; representing the economic and the noneconomic sectors, respectively) and in region *r*; $\Delta T_{t,r}$ is the increment in regional temperature with respect to its preindustrial value (in this case, its value in 1880); *β* is the exponent that determines the functional form of the impact function; and $\alpha_{d,r}$ are regional parameters to express the percentage of GDP ($Y_{t,r}$) lost for a benchmark warming of 2.5°C in each impact sector and region. Equation (6) represents the impacts associated to the occurrence of a large-scale discontinuity in the climate system. $D_{t,r}$ represents the economic impacts of a discontinuity at time *t* and region *r*; $\gamma_{t,r}$ is the economic impact of a discontinuity at time *t* in region *r*; and *π* is the probability of occurrence of the discontinuity. The total economic impacts are the sum of equations (5) and (6).

Given that the observed warming during the 20th century was below the lower limit for the occurrence of large-scale discontinuities, the economic damages presented here come from equation (5) only. The regional weights for scaling the impact functions are those from the PAGE2002 model (reproduced in S2 Table) and the regional estimates of temperature where produced using the scaling factors obtained from the emulation of the UKMOHADCM3 General Circulation Model of the Magicc/Scengen software (<http://www.cgd.ucar.edu/cas/wigley/magicc/>)[[1]](#footnote-2). The outcomes of the damage functions described above were estimated using simulation experiments of 1,000 realizations and the time-step was chosen to be one year. The global estimates of the climate damages during the 20th century presented here are simple averages of the regional damage functions.

**1.4. Damage function from recent literature review**

Based on a literature review of all published estimates of the global costs of climate change, Tol[9,10] estimates a damage function that synthesizes all findings. The damage function takes the same functional form of equation(4), but the parameters values are $\theta_{1}=\text{-0.25}$and $\theta_{2}=\text{-0.16}$. For this damage function the same temperature values as DICE were used. We will refer to this impact function as MA (for meta-analysis).

**2. The anthropogenic and natural components of the estimated impacts per sector.**

S2 Fig shows the anthropogenic and natural contributions to the economic costs of observed 20th century climate for agriculture, health, water resources, and energy obtained using FUNDn3.6. Agriculture is the sector for which the observed climate had the largest effect, leading to benefits of about 0.8% of GDP in 2000 (S2 Fig panel a). This sector is by far where the anthropogenic influence is more evident, leading at the end of the 20th century to gains of 0.68% of GDP. This is the only sector for which the anthropogenic contribution is considerably larger than the effects of natural variability. Carbon dioxide fertilization contributes most to these gains. The effects of natural forcing became positive around the 1930's and reached 0.06% in 2000, about one order of magnitude lower than the estimates of the anthropogenic contribution. For this sector, natural variability produced fluctuations in the range of about -0.15% to 0.06% of GDP, substantially larger than the contribution of natural forcing.

In the water resources sector, both anthropogenic and natural forcing imparted a trend on losses (S2 Fig panel b). The anthropogenic contribution led to losses of up to -0.12% of GDP and, in the last decades of the past century, it became about five times larger than the effects of natural forcing. However, the amplitude of the costs produced by low-frequency natural variability is considerably larger compared to the individual or joint contributions of natural and anthropogenic factors.

Low-frequency natural variability plays a dominant role on the costs of the two remaining sectors (S2 Fig panels c and d). The interaction effects between natural variability and forcing factors are large and add significant noise to the anthropogenic and natural forcings signals. In the energy sector benefits from the observed global temperature of about 0.36% were attained in 2000 (S2 Fig panel c). The anthropogenic forcing contributed to these gains during the whole 20th century reaching up to 0.20% in the 1990s. In comparison, the positive effects of natural forcing started around the 1930s and due to the interaction effects, the benefits from natural forcing reached 0.34% in 1990. In 1995, the anthropogenic and natural contributions generated gains of about 0.17% and 24%, respectively, and then dropped considerably. Although in all sectors the effects of the slowdown in the warming can be detected, in the energy sector this is more evident due to the large interaction effects of forcing factors and natural variability. In 2000 the benefits of anthropogenic and natural forcing amounted to only 0.02% and 0.08%, respectively.

In the health sector, the negative impacts of the 20th century climate reached about 0.2% of GDP in 2000 (S2 Fig panel d). Although the contribution of anthropogenic forcing was negative during most of the century it was not until the 1970s that a negative trend became noticeable. For this sector, both anthropogenic and natural contributions to the costs of the 20th century climate are well within the amplitude of the effects of natural variability.

Nevertheless, as shown in S3 Fig, the anthropogenic contribution to the estimated number of deaths per million people related to climate is dominant (panel b). As shown in panels a) and b) of S2 Fig, the trend in the estimated number of deaths of these of climate related diseases is mainly imparted by the anthropogenic forcing. The largest contribution of anthropogenic forcing to these numbers occurs in diarrhoea, respiratory diseases and malaria. Natural forcing (panel c) and internal variability (panel d) mainly provided the low-frequency oscillatory pattern shown by the proportion of deaths.

**References**

1. Anthoff D, Tol RSJ. Climate policy under fat-tailed risk: an application of FUND. Ann Oper Res. 2014;220: 223–237. doi:10.1007/s10479-013-1343-2

2. Tol RSJ. Estimates of the Damage Costs of Climate Change. Part 1: Benchmark Estimates. Environ Resour Econ. 2002;21: 47–73. doi:10.1023/A:1014500930521

3. Tol RSJ. Estimates of the Damage Costs of Climate Change, Part II. Dynamic Estimates. Environ Resour Econ. 2002;21: 135–160. doi:10.1023/A:1014539414591

4. Tol RSJ. The economic impact of climate change in the 20th and 21st centuries. Clim Change. 2013;117: 795–808. doi:10.1007/s10584-012-0613-3

5. Nordhaus WD. A question of balance: Weighing the options on global warming policies. Yale University Press; 2008.

6. Nordhaus WD, Boyer J. Warming the world: economic models of global warming. MIT press; 2003.

7. Nordhaus WD. Economic aspects of global warming in a post-Copenhagen environment. Proc Natl Acad Sci U S A. 2010;107: 11721–6. doi:10.1073/pnas.1005985107

8. Hope C. The marginal impact of CO2 from PAGE2002: An integrated assessment model incorporating the IPCC’s five reasons for concern. Integrated Assessment. 2006.

9. Tol RSJ. The Economic Effects of Climate Change. J Econ Perspect. 2009;23: 29–51. doi:10.1257/jep.23.2.29

10. Tol RSJ. Correction and Update: The Economic Effects of Climate Change †. J Econ Perspect. 2014;28: 221–226. doi:10.1257/jep.28.2.221

1. The regional scaling factors are: 1.56 for Europe; 1.39 for Latin America; 1.49 for North America/OECD; 1.30 for Africa; 2.04 for North Asia; 1.33 for South Asia and; 1.45 for China. [↑](#footnote-ref-2)
